# Validation and Reliability of a Novel Vagus Nerve Neurodynamic Test and Its Effects on Heart Rate in Healthy Subjects: Little Differences Between Sexes

**DOI:** 10.3389/fnins.2021.698470

**Published:** 2021-09-06

**Authors:** Giacomo Carta, Agnese Seregni, Andrea Casamassima, Manuela Galli, Stefano Geuna, Pasquale Pagliaro, Matteo Zago

**Affiliations:** ^1^Human Anatomy, Department of Biological and Clinical Sciences, University of Turin, Turin, Italy; ^2^Neuroscience Institute Cavalieri Ottolenghi (NICO), University of Turin, Turin, Italy; ^3^Department of Rehabilitation, Sesto Hospital, ASST Nord Milano, Milan, Italy; ^4^Department of Electronics, Information and Bioengineering (DEIB), Politecnico di Milano, Milan, Italy; ^5^General Surgery Department, S. Maria delle Stelle Hospital, ASST Melegnano e Martesana Melzo, Milan, Italy; ^6^Human Physiology, Department of Biological and Clinical Sciences, University of Turin, Turin, Italy; ^7^Department of Mechanics, Politecnico di Milano, Lecco, Italy

**Keywords:** vagus nerve stimulation, heart rate, diagnostic test, ultrasound, neuropathic pain

## Abstract

**Background:**

The vagus nerve (VN), also called the pneumogastric nerve, connects the brainstem to organs contained in the chest and abdomen. Physiologically, VN stimulation can rapidly affect cardiac activity and heart rate (HR). VN neuropathy can increase the risk of arrhythmias and sudden death. Therefore, a selective test of VN function may be very useful. Since peripheral neurodynamic tests (NDT) are reliable for the assessment of neuropathies in somatic nerves, we aimed to validate a novel NDT to assess VN activity, namely, the VN-NTD.

**Methods:**

In this cross-sectional double-blind, sex-balanced study, 30 participants (15 females) completed a checklist of autonomic dysfunction symptoms. During the VN-NDT administration, HR and symptoms (i.e., mechanical allodynia) were monitored in parallel to a real-time ultrasonography imaging (USI) and motion capture analysis of the neck. The VN-NDT impact on HR and its accuracy for autonomic symptoms reported in the last 7 days were tested.

**Results:**

The VN-NDT induced a significant HR reduction of about 12 and 8 bpm in males and females [*t*(1, 119) = 2.425; *p* < 0.017; η_p_^2^ = 0.047, 95% confidence interval (CI): 0.93–9.18], respectively. No adverse events were observed during VN-NDT. A substantial interexaminer agreement between the evaluators in symptoms induction by VN-NDT was detected [*F*(1, 119) = 0.540; *p* = 0.464; η_p_^2^ = 0.005, low effect]. Notably, mechanical allodynia accuracy for gastrointestinal dysfunctions was excellent (*p* < 0.05; 95% CI: 0.52–0.73; *p* < 0.001; 95% CI: 0.81–0.96).

**Conclusions:**

The novel VN-NDT is a valid and accurate test capable of detecting VN activation with high sensitivity. Data provided are suitable for both sexes as a hallmark of HR variation due to VN normal response. The proposed VN-NDT may be reliable as daily routine neurological examination tests for the evaluation of neuropathic signs related to neuroinflammation of the VN.

**Clinical Trial Registration:**

www.ClinicalTrials.gov, identifier NCT04192877.

## Introduction

Several health-related conditions have prominent and clinically important manifestations including autonomic peripheral neuropathies (APN) (Freeman, 2005). The main causes described in the literature are diabetes, amyloidosis, immune-mediated, neoplastic, paraneoplastic, hereditary, secondary to infectious diseases, and intoxications (Oaklander and Nolano, 2019). The pathophysiology of neurodegenerative disorders often involves a microbiota–gut–brain axis perturbation (Fung et al., 2017). Cranial neuropathies due to acute diseases like Zika virus-associated Guillain–Barré syndrome (Parra et al., 2016), local lesions like in schwannomas of the vagus nerve (VN) (Sunaryo et al., 2012), and iatrogenic damage of the VN can affect nerve function with critical immediate (bradycardia and cardiac asystole) or delayed consequences (Nazir et al., 2017; Aggarwal et al., 2018).

Autonomic neuropathies are conditions difficult to be detected that increase hemodynamic instability (Ang et al., 2020), postsurgery complications (Lankhorst et al., 2015; Suarez-Roca et al., 2019), and sudden death in obese and diabetic patients (Freeman, 2005; Santos Breder and Sposito, 2019; Williams et al., 2019; Malaty et al., 2021). Patients with coronavirus disease 2019 (COVID-19) have an increased prevalence of cardiac arrhythmias (Ho et al., 2020) with an estimated incidence of 15% in post-COVID-19 patients (Malaty et al., 2021). Since APN is a growing health problem, it is of paramount importance to have a reliable clinical tool that investigates selectively VN neuropathies. Ultrasound imaging (USI) is the most reliable and cost-effective imaging tool to assess VN morphology (Anil and Tan, 2011; Kasehagen et al., 2018), but morphological changes alone cannot predict the clinical conditions of patients. A “gold standard” to assess only the VN functioning level excluding its interaction with the sympathetic system is missing, so general autonomic response tests, involving sympathetic and parasympathetic responses, are used with no negligible side effects and risks like retinal detachment, syncope, chest pain, and arrhythmias (Valsalva maneuver, tilt-table protocols, lower body negative pressure, noradrenaline spillover, etc.) (Fujii et al., 2004; Schrezenmaier et al., 2007; Pstras et al., 2016; Fajgenbaum et al., 2018; Ehrman et al., 2020). Therefore, a selective and reliable test to assess VN functions with no or possibly negligible side effects is necessary. Peripheral nerve selective tension tests or neurodynamic tests (NDT) are bedside examinations and reliable clinical tests validated for the detection of neuropathies of the somatic nerves (Taenzer et al., 2000; Wasan et al., 2011; Bueno-Gracia et al., 2016; Verwoerd et al., 2016; Ekedahl et al., 2018; Koulidis et al., 2019). NDTs assess the nerve response to mechanical stimuli which are transduced by stretch-sensitive ion channels in peripheral nerves also present in the VN axons and cell body membranes (Beaulieu-Laroche et al., 2020; Bonet et al., 2021). Therefore, the aim of the present study was threefold: (1) to describe and validate a tool for selective VN assessment as NDT of the VN (VN-NDT), (2) to collect normative data to define a hallmark of physiological spectrum in males and females for heart rate (HR) variations induced by the VN-NDT maneuvers, and (3) to describe the relationship between symptoms induced during the VN-NDT and any autonomic dysfunction-related symptom.

## Materials and Methods

### Study Design

Since no selective test for the VN exists, a validation process was performed *ex novo* taking advantage of the available data reported in the literature. An *a priori* power analysis was performed referring to Cohen’s kappa coefficient values reported by Martínez-Payá et al. (2015) (*k* = 0.66; *k* = 0.94) studying the USI during a neurodynamic test. A sample size of 30 subjects provided a statistical power of 0.90 assuming a moderate strength of agreement between two evaluators and correct classification of subjects as positive of 0.50 with an alpha of 0.05. Also, considering an HR reduction induced by the test similar to the one described by Antonino et al. (2017) (η^2^ = 1.134), 13 subjects for each sex were identified to provide a statistical power of 0.96 with an alpha error of 0.05 and 1 − β error of 0.95. Estimating a 20% dropout rate, we enrolled 36 subjects in the study. An expert (a physical therapist with more than 12 years of experience in neurodynamic test administration) and a novice examiner (a medical doctor with no training in neurodynamic tests) blinded to their judgments performed the maneuver sequences of VN-NDT to every participant on the VNs of participants on both sides.

Participants voluntarily took part in the examination after an explanation of all the risks and benefits, and they all signed the written informed consent form according to the Declaration of Helsinki. Before data collection, the study was approved by the University Bioethics Committee (protocol 139870-14/03/2019) and registered on www.ClinicalTrials.gov (trial registration number: NCT04192877) on December 5, 2019. Subjects were enrolled from December 12, 2019^1^. Participants were asked to not consume tea, caffeine, energy drinks, alcohol, and tobacco within 2 h of the study and avoid them 24 h before the study. Subjects were blinded to the expertise level of the evaluators, and the results were communicated only when the assessment was completed. Also, USI during the VN-NDT was performed by an expert medical doctor, currently a licensed USI international instructor in critical and acute care. The test results were available to the participants and evaluators only at the end of the study.

### Settings

The study was conducted in the Posture and Movement Analysis Laboratory of the Department of Electronics, Information, and Bioengineering, Politecnico di Milano.

### Inclusion/Exclusion Criteria and Motivation

Subjects were included if they were between 18 and 70 years old and sober. Subjects were excluded if they reported significant neck pain or headache [with Numeric Pain Rating Scale (NPRS) greater than 3/10] (Salaffi et al., 2004), pregnancy, recent neck or cardiac surgery or significant trauma in the preceding 3 months, cancer or inflammatory disorders, spinal cord or cauda equina signs, widespread neurological disorders affecting the tone of the upper limb and neck muscles, or underlying diseases, such as diabetes mellitus.

### Procedures and Data Collection

Data collection was performed in a standardized order: (1) fulfillment of self-report questionnaires, (2) neurological examination, (3) VN-NDT under USI and motion capture analysis assessment (MCA), and (4) short-term autonomic response (STAR) measured based on HR.

#### Self-Report Questionnaires

Epidemiological data (Supplementary Table 1), diagnosis, medication prescribed, a checklist of AD symptoms, and signs were declared by every participant (Terkelsen et al., 2017). An 11-item Likert scale was also administered to assess the perceived health status (PHS: 100 the best, 0 the worst health status ever).

#### Neurological Examination

A segmental neurological examination was performed to confirm that the participants had no signs of nerve conduction loss. In short, dermatomes from C2 to C5 were evaluated bilaterally with a 10-g monofilament (Paisley et al., 2002). The presence of mechanical allodynia as a sign of central sensitization (Jensen and Finnerup, 2014) was assessed by asking the participants to keep a clothes peg on the middle fingernail for 5 s and on the middle earlobe (to assess sensitization away from the “assessed area”) of both sides (Egloff et al., 2011).

Sensory discrimination was tested by administering a random sequence of 10 nociceptive and tactile stimuli on the skin of the neck (using a Neuropen^®^, Owen Mumford Ltd., Woodstock, United Kingdom). The upper limb NDT (ULNDT) was administered bilaterally to assess any subclinical neuropathic condition involving the neck or upper limbs (Schmid et al., 2009).

Participants were instructed to verbally stop the test immediately when any type of tension, discomfort, or unpleasant sensation was felt during the sequence of passive movements of the VN-NDT. The location of the symptom and behavior were defined using a pain drawing tool at the end of every single test (Bertilson et al., 2007), and their intensity was rated (NPRS) (Salaffi et al., 2004).

#### Vagus Nerve Neurodynamic Test

**FIGURE 1:**
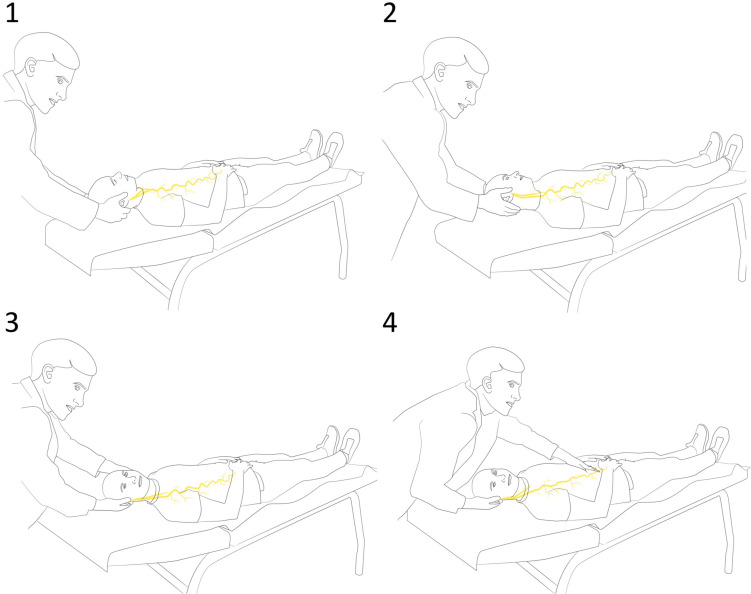
Sequence of the vagus nerve neurodynamic test (VN-NDT) with **(1)** starting position with upper cervical spine in flexion. **(2)** Contralateral lateral flexion. **(3)** Ipsilateral neck rotation. **(4)** The end position of the test with gentle movements of the upper abdomen caudally and cranially as discrimination maneuverers.

The VN emerges from the medulla of the brainstem and reaches the coeliac and mesenteric plexi in the abdomen passing through the jugular foramen (Verlinden et al., 2016) of the skull, between the internal carotid artery and the jugular vein in the neck and between the cardiac and pulmonary plexi in the thorax. The VN-NDT was developed starting from its morphology, selecting a combination of physiological movements that induce a higher mechanical tension on the nerve (Figure 1A). The subjects were assessed supine on an examination table, and evaluators were standing at the cranial short side of the table.

Upper cervical flexion and contralateral lateral flexion were selected for loading the intracranial part (Verlinden et al., 2016). Ipsilateral neck rotation was added to load the cervical tract.

Considering that the VN has afferent endings that are mechanosensitive (Zeng et al., 2018; Besecker et al., 2020), discrimination between VN and other tissues was performed while holding the head of the subject in the final pose, gently pushing the upper abdomen caudally and cranially to load and unload the thoracic tract. The test was positive (indicating abnormal responses) if discrimination maneuvers changed the symptoms of the subject indicating a neurogenic source; otherwise, it was declared negative (Schmid et al., 2009). To standardize the test, all participants were placed in the supine position without a pillow in a room at 25°C for 30 min as described by Fujii et al. (2004).

#### Short-Term Autonomic Response

Short-term autonomic response was assessed as described by Devalle and coworkers, which is a reliable outcome for the autonomic response to pain even in subjects with disturbances of consciousness, comparing the HR values (fingertip portable pulse oximeter Intermed SAT-200) at rest (10 s after the ULNDT administration) and after a 10-s window holding the end position of the VN-NDT (Devalle et al., 2018). Moreover, we verified that no chances in HR were induced by abdominal compression alone. To avoid any placebo/nocebo response, HR was blinded to the assessors and participants.

### Ultrasound Imaging and Motion Capture Analysis Protocols

Protocols defined by Martinoli and coworkers for the detection of the anterior tubercle of C6 (Martinoli et al., 2002) and the cervical tract of the VN (Giovagnorio and Martinoli, 2001) were adopted. Participants were assessed in a supine position on a medical table and real-time USI was performed by the medical doctor standing near to the right long edge of the table, while the assessor performing the neurodynamic test was standing near to the short edge of the table where the head of the participant was (Figure 1 and Supplementary Figure 1). Axial scans were obtained using the inferior margin of the thyroid as an initial reference from which the probe was moved laterally to the region of the transverse processes. The probe was moved cranially till the anterior tubercle of C6 was detected. Distance between the VN and C6 anterior tubercle (VN–C6) was measured at rest and at the final position of the VN-NDT to quantify the lateralization or proximalization of the VN induced by the test, suggesting an increased or decreased tension on the wire-like structure of the VN. Esaote^®^ MyLab Alpha (Esaote S.p.A, Genoa, Italy) USI equipment was used with a 5–7-MHz convex array probe (Figure 1B). All subjects were screened for thyroid problems at the end of the assessment.

Throughout the whole duration of the VN-NDT and real-time USI assessment, the three-dimensional head orientation of the subjects was recorded at 100 Hz with an optoelectronic motion capture system (Smart-DX, BTS S.p.A., Milan, Italy). A cluster with three retroreflective markers (diameter: 15 mm) was secured on the head of the subject using an elastic band; three additional markers were fixed on the acromion and the sternum (Supplementary Figure 1). The rest and final head positions were manually annotated upon explicit communication by the USI operator. System calibration was conducted according to the guidelines of the manufacturers and returned an average error in marker position of 0.35 mm, on a working volume of 2.6 × 1.8 × 2.5 m^3^.

### Data Analysis

Differences from baseline were checked, as well as the effects between and within factors among symptoms induced by the test and perceived AD signs and symptoms. Custom routines were developed within Smart Analyzer (version 1.10.465, BTS S.p.A) to extract kinematic data. Three-dimensional coordinates were smoothed with a fourth-order low-pass Butterworth filter with a cutoff frequency of 1 Hz. A local reference system fixed on the head was defined: the *x*-axis was anteroposterior and pointed forward; the *y*-axis was craniocaudal and pointed upward; the *z*-axis was mediolateral and pointed to the right of the subject. The acromial and sternum markers defined a local trunk coordinate system (Zago et al., 2020), with an analogous axes convention, that served as a reference for head orientation (Supplementary Figure 1).

Head lateral inclination on the frontal plane (positive to the right), axial rotation on the transverse plane (positive to the left), and flexion (negative)–extension (positive) angles on the sagittal plane were computed as the Euler angles (*XYZ* rotation sequence) between the head and trunk reference frames, respectively. The initial position, i.e., that assumed by the participants laying down on the bed before the test initiation, was taken as neutral (all angles equal to zero). An explanatory representation of head rotations during the test is depicted in Figure 4A.

**FIGURE 2:**
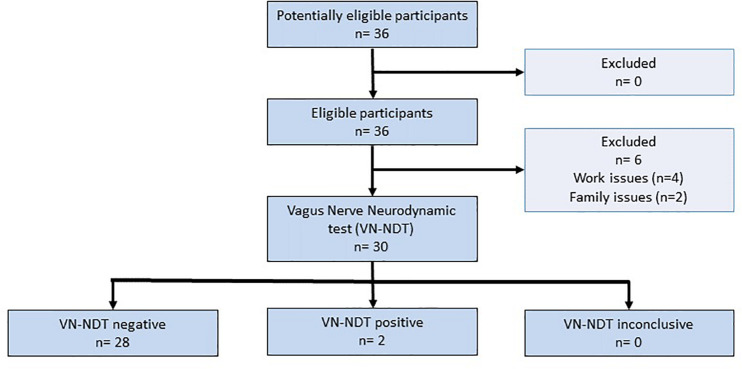
STARD flowchart of the vagus nerve neurodynamic test (VN-NDT).

**FIGURE 3:**
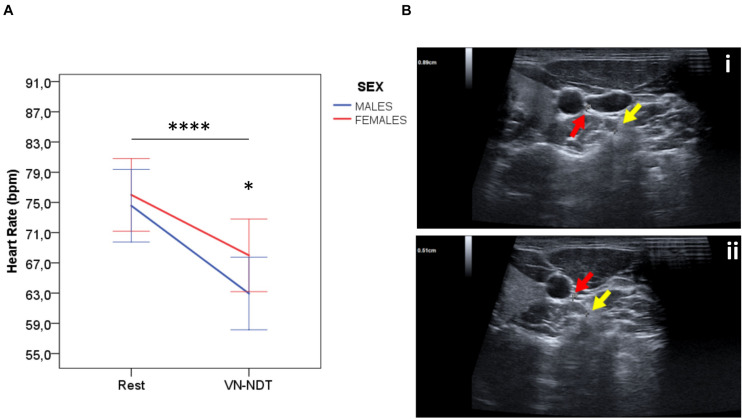
**(A)** Effect of the VN-NDT on HR in males and females. Values in the graph are expressed as mean ± SD. Two-way ANOVA was carried out (data are normally distributed with comparable variances); asterisk shows the statistically significant difference between sexes (*p ≤ 0.05 and ****p ≤ 0.0001). **(B)** Ultrasound imaging axial scans of the (i) right vagus nerve at rest and (ii) the final position of the neurodynamic test. The red arrow indicates the vagus nerve and the yellow arrow indicates the anterior tubercle of C6 in male or female participants (no differences between sexes were detected by USI, *p* = 0.54).

**FIGURE 4:**
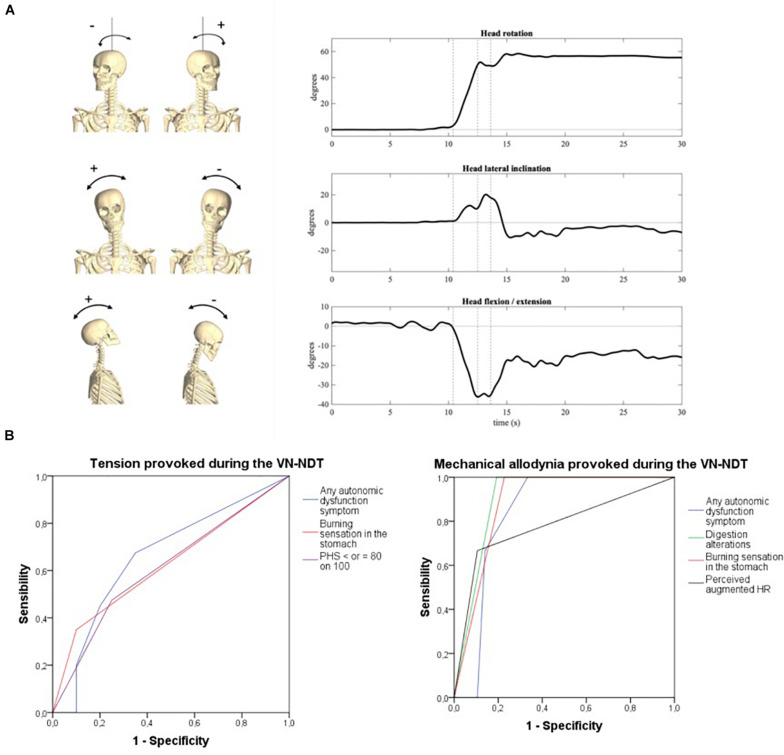
**(A)** Head orientation at the end of the neurodynamic test (R: head inclined on the right of the subject; L: head inclined on the left of the subject). (i) Inclination, (ii) rotation, and (iii) flexion/extension. The three dotted vertical lines in the graphs represent the three movements of the VN-NDT combined from the left to the right: upper cervical flexion, contralateral later flexion, and ipsilateral rotation, respectively. **(B)** ROC curves of symptoms related to vagal dysfunctions or autonomic peripheral neuropathies detected by the onset of tension (left) and pain (right) in the neck during the vagus nerve neurodynamic test. PHS, perceived health status; HR, heart rate.

To provide an indirect measure of the vagal strain level, we also measured the distance between the sternum and right (or left) head marker, according to the side the test was performed on. The ratio between final and initial values was termed as head displacement ratio: higher values indicate larger head motion and vagal strain.

### Statistics

Statistical analyses were performed within SPSS v.20.0 (IBM Corp., Armonk, NY, United States). Paired Student’s *t*-tests were used to detect differences from rest to end position in terms of STAR and VN–C6 distance. As an effect size measure, Cohen’s *d* was used. The agreement in reporting test outcomes between the two operators was computed as Cohen’s kappa (Martínez-Payá et al., 2015).

Receiver operating characteristic (ROC) curves were adopted to define the sensibility, specificity, and positive and negative likelihood ratios of the VN-NDT-related symptoms to predict VN dysfunctions or neuropathies. The overall diagnostic accuracy of the VN-NDT was defined by the area under the curve (AUC); a value of 0.5 was deemed as no discrimination, a value from 0.7 to 0.8 as acceptable, from 0.8 to 0.9 as excellent, and more than 0.9 as outstanding (Sarkar and Midi, 2010). CI at 95% was calculated and a statistical significance level of 0.05 was implemented throughout.

A two-way analysis of variance (ANOVA) for repeated measures with a 2 × 2 full-interaction design was adopted to test changes on the side (test administered on the right or left of the participant) and operator factors (experienced, not experienced) on the following variables: tests positivity, symptoms location, anatomical and physiological parameters assessed at rest and end of the VN-NDT, angular rotations, and head displacement ratio. The two-way ANOVA for repeated measures was also adopted to define differences between sexes and HR variations induced by the VN-NDT test. The effect size of each factor was computed as partial eta-squared (η_p_^2^): a value of η_p_^2^ of 0.010 was considered a small effect, a value of 0.059 a medium effect, and a value of 0.138 a large effect (Richardson, 2011).

## Results

As can be seen in Table 1, 46.7% of the participants had at least one symptom of the AD checklist (nine females and six males); 23.3% of the subjects had experienced in the previous 7 days an episode of orthostatic hypotension (five females and two males), and one-fourth reported gastrointestinal symptoms (six females and three males).

**TABLE 1:** Differences between sexes at baseline characteristics and reported autonomic signs and symptoms (experienced during the last 7 days).

Variable	*F*	*M*	Total/cases (%)	*p*
**Epidemiologic data**				
Age, years	31.68 ± 11.08	31.64 ± 13.44	31.7 ± 12.0	0.99
Education	Bachelor’s degree	Bachelor’s degree	Bachelor’s degree	0.15
Smoke	0	0.14 ± 0.36	2 (6.7)	0.13
BMI	22.4 ± 3.37	23.2 ± 2.37	22.8 ± 2.92	0.45
NRS (0–10 points)	0.66 ± 1.19	1.1 ± 1.62	0.87 ± 1.4	0.39
Health status (0–100 points)	82.81 ± 13.9	87.14 ± 12.04	84 ± 13	0.38
HR at rest (bpm)	76 ± 12.22	74.57 ± 11.59	75.3 ± 11.7	0.75
**Autonomic checklist**				
At least one autonomic symptom	0.56 ± 0.51	0.36 ± 0.51	14 (46.7)	0.28
Nausea	0.12 ± 0.34	0	2 (6.7)	0.18
Orthostatic hypotension	0.31 ± 0.48	0.14 ± 0.36	7 (23.3)	0.29
Digestion alterations	0.32 ± 0.48	0.33 ± 0.48	7 (23.3)	0.29
Breathing alterations (shortness of breath)	0	0	0	–
Voice changes	0	0	0	–
Altered deglutition	0.06 ± 0.25	0	1 (3.3)	0.36
Perceived augmented HR	0.19 ± 0.4	0.07 ± 0.27	4 (13.3)	0.37
Perceived reduced HR	0.06 ± 0.25	0	1 (3.3)	0.36
Burning sensation in the stomach	0.25 ± 0.45	0.28 ± 0.47	8 (26.7)	0.83
Constipation	0.06 ± 0.25	0	1 (3.3)	0.36
Diarrhea	0.06 ± 0.25	0	1 (3.3)	0.36
Vomiting	0.06 ± 0.25	0	1 (3.3)	0.36
Augmented lacrimation	0	0	0 (0)	–
Reduced lacrimation	0.12 ± 0.34	0	2 (6.7)	0.183
Augmented salivation	0	0	0 (0)	–
Reduced salivation	0	0	0 (0)	–
Head and neck sweating attacks	0.06 ± 0.25	0	1 (3.3)	0.36
Head and neck skin dryness	0.06 ± 0.25	0	1 (3.3)	0.36
Sleep alteration	0.34 ± 0.48	0.14 ± 0.36	7 (23.3)	0.29

*Numeric values are reported as means ± standard deviation (SD); nominal values are reported as medians. The checklist was administered to all participants and answers are expressed as the number of subjects having symptoms and percentage (%).*

*HR, heart rate; AD, autonomic dysfunction; APN, peripheral neuropathies; *p*, level of significance (*p* < 0.05).*

The STARD flowchart (Figure 2) shows that six out of the 36 participants were not able to perform the experiments because of work or family issues. Notably, the sample size actual power was not affected by the loss of participants since 20% of the dropout was calculated as reported above. The age of the participants was not significantly different between males and females [*t*(1, 30) = −0.01; *p* = 0.992; 95% CI: −9.22 to 9.21]. Twenty-one out of 30 subjects were pain-free, five reported low back pain, three leg pain, and one facial pain. Five participants had a medical diagnosis with drug prescriptions: two for asthma, one for hyperthyroidism, one for gastric reflux, and one for hypertension and gastric reflux. No alteration was detected at the neurological examination for all participants. Four subjects reported mechanical allodynia of the right ear lobe. The ULNDT was positive on both sides in two subjects and on one side in four subjects. Cohen’s kappa of 0.67 (95% CI: 0.49–0.85; *p* < 0.001) defined that VN-NDT reliability was significantly substantial.

No significant differences were detected between the sides and positive or negative tests between the two evaluators (Supplementary Table 1) nor the type and location of symptoms provoked [*F*(1, 119) = 0.540; *p* = 0.464; η_p_^2^ = 0.005, low effect]. Tension or mechanical allodynia in the suboccipital ipsilateral neck portion was reported in 66.7 and 5% of the cases, respectively. No adverse events (nausea, vomiting, hypotension, or neurological symptoms) were recorded during and after the VN-NDT administration.

The HR of the participants (Figure 3A) at rest (75.33 ± 11.61 bpm, *n* = 30) displayed no significant differences between females and males [*t*(1, 119) = −0.672; *p* = 0.502; η_p_^2^ = 0.004, 95% CI: −5.64 to 2.78]. Intriguingly, the VN-NDT induced a significant HR reduction in all participants [*F*(1, 119) = 89.919; *p* < 0.000; η_p_^2^ = 0.432, very high effect]. The HR drop was of 8 (±12.13) in females and 11.63 (±10.02) bpm in males and resulted statistically different between females and males [*t*(1, 119) = 2.425; *p* = 0.017; η_p_^2^ = 0.047, 95% CI: 0.93–9.18]. Notably, even when mild pain was provoked, an HR reduction was recorded confirming a selective VN stimulation by the VN-NDT (Devalle et al., 2018).

### Anatomical and Biological Variables

The USI revealed no variations nor pathologies of the cervical portion of the VN (Giovagnorio and Martinoli, 2001). The VN-NDT induced a significant overall reduction of the VN–C6 distance (Figure 3B) of about 0.1 mm [*t*(1, 119) = 2.48; *p* < 0.01; *d* = 0.2; 95% CI: 0.03–0.3]. The VN–C6 distance was significantly higher on the right side at rest and in the VN-NDT end position of 0.30 and 0.34 mm, respectively [*t*(1, 118) = 3.24; *p* < 0.002; *d* = 0.592; *t*(1, 118) = 3.83; *p* < 0.000; *d* = 0.699, respectively]. USI identified a significant interaction (Supplementary Table 1) for side factor [*F*(1, 119) = 14.98; *p* < 0.000; η_p_^2^ = 0.114] and between operator but not for side factor and VN–C6 distance [*F*(1, 119) = 0.032; *p* = 0.571; η_p_^2^ = 0.003]. Also, no significant interaction between sexes of the participants and VN–C6 changes before and after the test was detectable [*F*(1, 119) = 0.378; *p* = 0.540; η_p_^2^ = 0.003]. These data indicate a higher distance on the right side between C6 and VN, but the degree of tension induced by the VN-NDT is similar to each side and not dependent on the sex of the participants.

### Head Kinematics

To reach the VN-NDT final position, the neck of the subject was moved to stretch one VN each time, from the anatomical position of rest, of about 52° (±11°) of ipsilateral to the tested side rotation, 12° (±8.5°) of contralateral lateral flexion, and 12° (±8.5°) of flexion (Figure 4A), which indicates that the test was performed in a normal cervical range of motion not able to overstress muscle ligaments and joints of this anatomical region. Neither head inclination nor head flexion–extension significantly changed between sides relative to the assessor factor (Supplementary Table 3). Conversely, the head was more laterally rotated by about 4° [*F*(1, 119) = 6.29; *p* = 0.015; η_p_^2^ = 0.101] when the left side was tested, as prompted in Supplementary Figure 2 and Supplementary Table 3. Consistently, a slightly but significantly higher head displacement ratio was observed on the left side [Supplementary Figure 2D, *F*(1, 119) = 6.211; *p* = 0.016; η_p_^2^ = 0.1, medium-to-large effect], especially when the novice assessor performed the test [*F*(1, 119) = 6.969; *p* = 0.011; η_p_^2^ = 0.111, medium-to-large effect]. No significant side-by-operator interaction was found.

### Autonomic Symptoms Detection Accuracy

The onset of tension or mechanical allodynia in the suboccipital ipsilateral region during the VN-NDT showed a significant ability to detect AD-related symptoms (Figure 4B and Supplementary Table 3). In particular, burning sensation in the stomach was significantly detected by tension and mechanical allodynia in the neck with an accuracy of 0.62 and 0.89, respectively (*p* < 0.026; 95% CI: 0.52–0.73; *p* < 0.001; 95% CI: 0.81–0.96; Table 2). Levels of PHS inferior or equal to 80 on 100 were significantly detected by neck tension (Figure 4B) with an accuracy of 0.61 (*p* < 0.045; 95% CI: 0.51–0.72).

**TABLE 2:** The vagus nerve neurodynamic test accuracy.

	**Tension**				**Pain (mechanical allodynia)**			
**Variable**	**Sensitivity**	**Specificity**	**+LR**	**−LR**	**Sensitivity**	**Specificity**	**+LR**	**−LR**
Digestion alterations					1	0.81	5.26	0
Perceived augmented HR					0.67	0.90	6.7	0.37
Burning sensation in the stomach	0.35	0.90	3.5	0.72	1	0.77	4.35	0
Any APN symptoms	0.67	0.65	1.91	0.51	1	0.46	1.85	0
Number of symptoms								
More than 1	0.45	0.80	2.2	0.7	1	0.67	3.03	0
More than 7	0	0.90	0	1.1	0	0.96	0	1.04
PHS (80 < on 100)	0.47	0.75	1.88	0.71				

*Results about symptoms-induced diagnostic features in detecting vagal impairment and APN-related symptoms of the vagus nerve neurodynamic test are reported above (only significant predicted symptoms using ROC curves were reported). Sensitivity, specificity, and positive and negative likelihood ratios are reported (+LR, −LR).*

*HR, heart rate; PHS, perceived health status.*

## Discussion

This study indicates that the proposed VN-NDT induces a consistently moderate HR reduction in subjects of both sexes. Therefore, we propose it as a sensitive, fast, and riskless screening test for vagal function assessment which could be useful in the assessment of autonomic nervous system neuropathies.

Our data validate the proposed VN-NDT as a selective tool for VN function assessment. The collected normative data define the hallmark of physiological spectrum in males and females for HR variations induced by the VN-NDT and suggest a relationship between symptoms induced during the test and some autonomic dysfunction-related symptoms.

As described by Velten et al. (2020), autonomic symptoms related to orthostatic hypotension are commonly reported in 20% of the healthy population. Indeed, none of the participants had a diagnosis related to an autonomic disease, but many had experienced 1 week before the test at least one symptom related to autonomic dysregulation. In particular, orthostatic hypotension and altered digestion were the more prevalent conditions. The VN-NDT induces an HR reduction greater than those reported with Valsalva maneuver (VM) (Schrezenmaier et al., 2007), VN transcutaneous, or direct electrical stimulation (Clancy et al., 2014; Anand et al., 2020). Indeed, the VN-NTD induces a consistent and significant HR reduction of about 8 bpm in females and 12 bpm in males, respectively, likely triggered by the stretch-sensitive baroreceptor fibers traveling in the nodose and petrosal sensory ganglia of the VN (Berthoud and Neuhuber, 2000; Zeng et al., 2018; Norcliffe-Kaufmann, 2019; Besecker et al., 2020). Although neck torsion during the test was performed in a physiological mid-range of motion and the hands of the assessor were positioned on the head and upper cervical spine of the participant, we cannot exclude a role for the esophageal intraganglionic laminar endings in mechanical stress transduction (Zagorodnyuk and Brookes, 2000; Brookes et al., 2013). A somewhat similar effect on HR has been found in normotensive humans during prolonged submaximal mandibular extension (60% of the maximal interincisal distance), prevented by minimal mandibular extension keeping a wooden tongue depressor between the incisors (Del Seppia et al., 2016, 2017). We cannot definitively rule out that similar effects are triggered by the two maneuvers, but the VN-NDT maneuvers did not induce any remarkable changes in the temporomandibular joint, prevented by the upper cervical flexion. Also, the effects on HR were detected at a short latency of 10 s of test administration, while the effects of the prolonged mandibular extension were recorded after 10 min of submaximal mandibular extension (Del Seppia et al., 2016; Devalle et al., 2018). Considering those data, we can reasonably hypothesize a marginal role of the glossopharyngeal nerve stretch reflex enrolment in the VN-NDT cardiac effects.

The VN-NDT is less invasive than the VM and other neural provocative tests (Schrezenmaier et al., 2007; Ehrman et al., 2020), since no side events were recorded, and no stress is applied to the cardiocirculatory system (Pstras et al., 2016). Also, no active participation of the tested subjects is required, which is particularly useful in subjects with communication problems like in the case of intensive care patients with COVID-19 and with disturbances of consciousness. The USI and the motion capture analysis confirmed that the VN-NDT induces a standardized anatomical reduction of the bone–nerve distance, which can stretch the VN and provoke symptoms related to autonomic dysfunctions.

The test accuracy and interrater agreement are comparable or higher than other clinical tests commonly used in the neurological assessment for neuropathic conditions like sensory testing, manual muscle testing, and nerve mechanosensitivity (Schmid et al., 2009; Terkelsen et al., 2017; Reshef et al., 2019).

Notably, mechanical allodynia—which is a common symptom when nerves receive prolonged exposure to inflammatory cytokines (Jensen and Finnerup, 2014; Beaulieu-Laroche et al., 2020; Bonet et al., 2021)—provoked by the VN-NDT had the best test accuracy in detecting digestion alterations and burning sensation in the stomach. Indeed, gastrointestinal dysfunctions are very common in acute and chronic APNs (Freeman, 2005; Oaklander and Nolano, 2019; Gutierrez et al., 2020; Marathe et al., 2020). Since the perioperative and postsurgery risks of cardiovascular side events (Lankhorst et al., 2015; Ho et al., 2020; Malaty et al., 2021) are higher in post-COVID-19 patients and patients with APN, which are difficult to be studied, it is possible to adopt the VN-NDT as a sensitive, faster, and riskless screening test. Yet, the test does not require other instruments than a finger pulse oximeter and a medical examination table, which makes it usable in low- and high-income countries.

Here, we report for the first time that a sequence of neck movements can systematically affect HR, both in males and females, suggesting a key role of the stretch on the neck portion of the VN in HR modulation. Gutierrez and coworkers reported that a patient with acute sensory and autonomic neuropathy had her symptoms relieved by neck movements (Gutierrez et al., 2020) which are included in the VN-NDT. Therefore, we can argue that studying the VN-NDT effects can be helpful in diagnosis and symptoms management in autonomic dysfunctions. Indeed, neurodynamic tests had been adopted successfully as treatment interventions for peripheral neuropathies. For instance, it has been established that invasive and non-invasive stimulation on the cervical tract of the VN ameliorates survival rates in sepsis models (Huston et al., 2007) and promotes heart and lung regeneration in preclinical models (Brandt et al., 2019; Chen et al., 2020), HR variability in cardiological patients (Kobayashi et al., 2013), and symptoms improvement in people with pharmacoresistant problems such as acute and chronic pain, dementia, psychiatric illness, consciousness disorder, and epilepsy (Kirchner et al., 2000; Schachter, 2006; Corazzol et al., 2017; Breit et al., 2018; Dong and Feng, 2018; Johnson and Wilson, 2018).

Since the VN-NDT can induce an effective VN stimulation, it would be useful to investigate its effects on these pathophysiological conditions and other conditions like diabetes-related gastrointestinal alterations, cardiac neuropathies, and arrhythmias secondary to coronavirus infection (Garamendi-Ruiz and Gómez-Esteban, 2019; Santos Breder and Sposito, 2019; Malaty et al., 2021).

## Conclusion

The tests currently available for APN are neither selective nor sex-specific for evaluating the parasympathetic nervous system and can have troubling side effects. The proposed VN-NDT is a reliable, sensible, and sustainable screening test to assess parasympathetic activity and VN alterations also in patients with verbal/communication problems. The physiological HR changes induced by the VN-NDT are provided for healthy males and females. The VN-NDT can be safely incorporated into bedside assessment routines and pretreatment routine tests for all conditions in which APN is suspected and to discriminate APN from neck musculoskeletal problems.

## Data Availability Statement

The raw data supporting the conclusions of this article will be made available by the authors, without undue reservation.

## Ethics Statement

The studies involving human participants were reviewed and approved by University Bioethics Committee (protocol 139870-14/03/2019). The patients/participants provided their written informed consent to participate in this study.

## Author Contributions

GC and MZ devised the concept of the study. GC, MZ, and AS designed the study. GC, MZ, AS, AC, MG, and SG participated in the acquisition of data. GC, MZ, AS, and PP analyzed and interpreted the data. GC, PP, and MZ wrote the initial draft. All authors critically revised the manuscript and approved the final version.

## Conflict of Interest

The authors declare that the research was conducted in the absence of any commercial or financial relationships that could be construed as a potential conflict of interest.

## Publisher’s Note

All claims expressed in this article are solely those of the authors and do not necessarily represent those of their affiliated organizations, or those of the publisher, the editors and the reviewers. Any product that may be evaluated in this article, or claim that may be made by its manufacturer, is not guaranteed or endorsed by the publisher.

## Footnotes

1
https://bit.ly/3kkhZQL
