# Supplementary material for: Validation and Reliability of a Novel Vagus Nerve Neurodynamic Test and Its Effects on Heart Rate in Healthy Subjects: Little Differences Between Sexes
Source: Front Neurosci. 2021 Sep 6;15:698470. doi: 10.3389/fnins.2021.698470 (PMC8450330; doi:10.3389/fnins.2021.698470)

Supplementary Material

**Table S-1:** ANOVA results of vagus nerve neurodynamic test, anatomical and physiological parameters (η_p_^2^:partial eta-squared).

|  | **Side factor** | | | **Operator factor** | | | **Side** $\times$ **Operator** | | |
| --- | --- | --- | --- | --- | --- | --- | --- | --- | --- |
| **Variable** | **F** | **p** | η_p_^2^ | **F** | **p** | η_p_^2^ | **F** | **p** | η_p_^2^ |
| VN-NDT results | 2.158 | 0.145 | 0.018 | 2.158 | 0.145 | 0.018 | 0.540 | 0.464 | 0.005 |
| Sensation of tension | 0 | 1.000 | 0 | 2.367 | 0.127 | 0.020 | 0 | 1.000 | 0 |
| Mechanical allodynia | 0 | 1.000 | 0 | 0.682 | 0.410 | 0.006 | 0 | 1.000 | 0 |
| Anatomical and physiological parameters before and after VN-NDT administration | | | | | | | | | |
| C6-VN distance at rest in mm (USI) | 10.37 | **0.002** | 0.082 | 0.203 | 0.653 | 0.002 | 0.273 | 0.602 | 0.002 |
| C6-VN distance at end of range in mm (USI) | 14.98 | **0.000** | 0.114 | 0.229 | 0.633 | 0.002 | 4.220 | **0.042** | 0.035 |
| HR at rest (beats per minute) | 0.617 | 0.434 | 0.005 | 0.022 | 0.883 | 0.000 | 0.177 | 0.895 | 0.000 |
| HR at end of range (beats per minute) | 0.617 | 0.434 | 0.005 | 0.022 | 0.883 | 0.000 | 0.017 | 0.895 | 0.000 |
| Ultrasonography imaging (USI); Heart Rate (HR) | | | | | | | | | |

**Table S-2:** Receiver Operating Characteristic (ROC) Curve for symptoms induced by the vagus nerve neurodynamic test.

|  | **Tension** | | | | **Pain** | | | |
| --- | --- | --- | --- | --- | --- | --- | --- | --- |
| **Variable** | **AUC** | **Std. Error** | **p** | **95% CI** | **AUC** | **Std. Error** | **p** | **95% CI** |
| Orthostatic Hypotension | 0.600 | 0.053 | 0.075 | 0.50–0.70 | 0.377 | 0.095 | 0.312 | 0.19–0.56 |
| Nausea | 0.475 | 0.057 | 0.656 | 0.36–0.59 | 0.465 | 0.114 | 0.773 | 0.24–0.69 |
| Digestion Alterations | 0.600 | 0.053 | 0.075 | 0.50–0.70 | **0.904** | 0.032 | **0.001** | 0.84–0.98 |
| Breathing Alterations (shortness of breath) | 0.500 | 0.056 | 1.00 | 0.39–0.61 | 0.500 | 0.121 | 1.00 | 0.26–0.74 |
| Voice Changes | 0.500 | 0.056 | 1.00 | 0.39–0.50 | 0.500 | 0.121 | 1.00 | 0.26–0.74 |
| Altered deglutition | 0.525 | 0.055 | 0.656 | 0.42–0.63 | 0.482 | 0.118 | 0.885 | 0.25–0.71 |
| Perceived augmented HR | 0.525 | 0.055 | 0.656 | 0.42–0.63 | **0.781** | 0.116 | **0.021** | 0.55–1.0 |
| Perceived reduced HR | 0.525 | 0.055 | 0.656 | 0.42–0.63 | 0.482 | 0.118 | 0.885 | 0.25-0.71 |
| Burning sensation in the stomach | **0.625** | 0.052 | **0.026** | 0.52–0.73 | **0.886** | 0.036 | **0.001** | 0.81–0.96 |
| Constipation | 0.450 | 0.058 | 0.373 | 0.34–0.56 | 0.482 | 0.118 | 0.885 | 0.25–0.71 |
| Diarrheal | 0.525 | 0.055 | 0.656 | 0.42–0.63 | 0.482 | 0.118 | 0.885 | 0.25–0.71 |
| Vomit | 0.450 | 0.058 | 0.373 | 0.34–0.56 | 0.482 | 0.118 | 0.885 | 0.25–0.71 |
| Augmented lacrimation | 0.500 | 0.056 | 1.00 | 0.39–0.61 | 0.500 | 0.121 | 1.00 | 0.26–0.74 |
| Reduced lacrimation | 0.400 | 0.058 | 0.075 | 0.29–0.51 | 0.465 | 0.114 | 0.773 | 0.24–0.69 |
| Augmented salivation | 0.500 | 0.056 | 1.00 | .039–0.61 | 0.500 | 0.121 | 1.00 | 0.26–0.79 |
| Reduced salivation | 0.500 | 0.056 | 1.00 | 0.39–0.61 | 0.500 | 0.121 | 1.00 | 0.26–0.74 |
| Head and neck sweating attacks | 0.525 | 0.055 | 0.656 | 0.42–0.63 | 0.482 | 0.118 | 0.885 | 0.25–0.71 |
| Head and neck skin dryness | 0.450 | 0.058 | 0.373 | 0.38–0.56 | 0.482 | 0.118 | 0.885 | 0.25–0.71 |
| Sleep alteration | 0.488 | 0.056 | 0.824 | 0.38–0.60 | 0.377 | .095 | .312 | 0.19–0.56 |
| Any APN symptoms | **0.661** | 0.055 | **0.004** | 0.55–0.77 | **0.839** | 0.040 | **0.005** | 0.76–0.92 |
| Number of symptoms | **0.638** | 0.054 | **0.014** | 0.53–0.74 | **0.746** | 0.068 | **0.043** | 0.61–0.88 |
| PHS (80< on 100) | 0.613 | 0.054 | **0.045** | 0.51–0.72 | 0.465 | 0.119 | 0.773 | 0.23–0.71 |
| Area under the Curve (AUC). Heart Rate (HR); Perceived health status (PHS) | | | | | | | | |

**Table S-3:** ANOVA results of vagus nerve neurodynamic test, head kinematics (η_p_^2^: partial eta-squared).

|  | **Side factor** | | | **Operator factor** | | | **Side** $\times$ **Operator** | | |
| --- | --- | --- | --- | --- | --- | --- | --- | --- | --- |
| **Variable** | **F** | **p** | **η_p_^2^** | **F** | **p** | **η_p_^2^** | **F** | **p** | **η_p_^2^** |
| Head orientation | | | | | | | | | |
| Inclination | 1.729 | 0.194 | 0.030 | 0.495 | 0.485 | 0.022 | 0.390 | 0.535 | 0.007 |
| Rotation | 6.288 | **0.015** | 0.101 | 2.692 | 0.106 | 0.046 | 0.045 | 0.832 | 0.001 |
| Flexion-extension | 0.718 | 0.400 | 0.012 | 0.000 | 0.984 | 0.001 | 1.295 | 0.260 | 0.022 |
|  | | | | | | | | | |
| Displacement ratio | 6.211 | **0.016** | 0.100 | 6.969 | **0.011** | 0.111 | 0.165 | 0.686 | 0.003 |

**Figure S1:Reference points for markers using the Smart-DX measuring system are reported**. On the trunk,markers were positioned at the following bony landmarks: acromion and manubrium sterni; on the head, a three-marker cluster was used. Real-time ultrasound imaging of the vagus nerve anatomical position was used during the neurodynamic test.


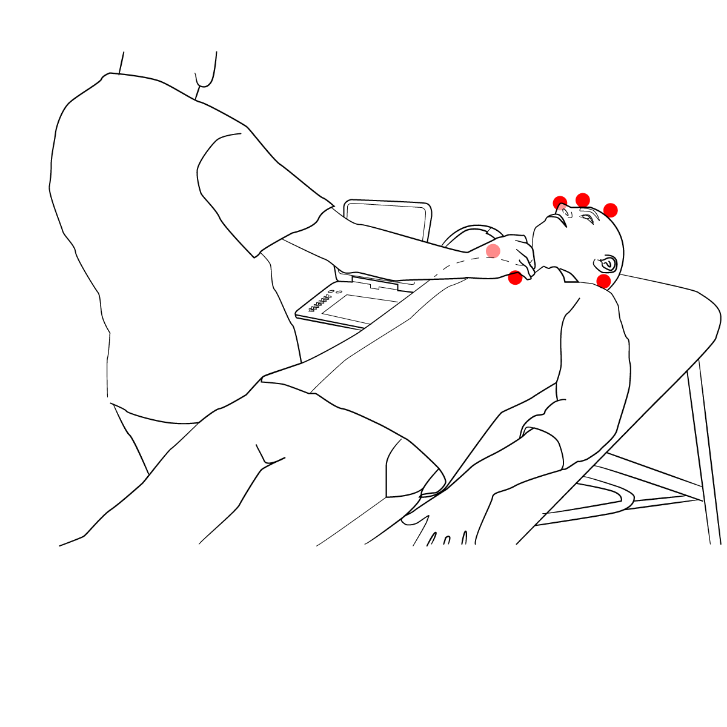


**Figure S2:Sequence of head and neck movements during the vagus nerve neurodynamic test.**

Head orientation at the end of the neurodynamic test (A) flexion/extension (B) inclination(C) rotation(L: left vagus nerve tested; R: right vagus nerve tested).(D)Head displacement ratio: the distance between the sternum and the ipsilateral head marker to the vagus nerve tested before and after the neurodynamic test is reported in the graph and a higher value indicates a larger strain of the vagal nerve.


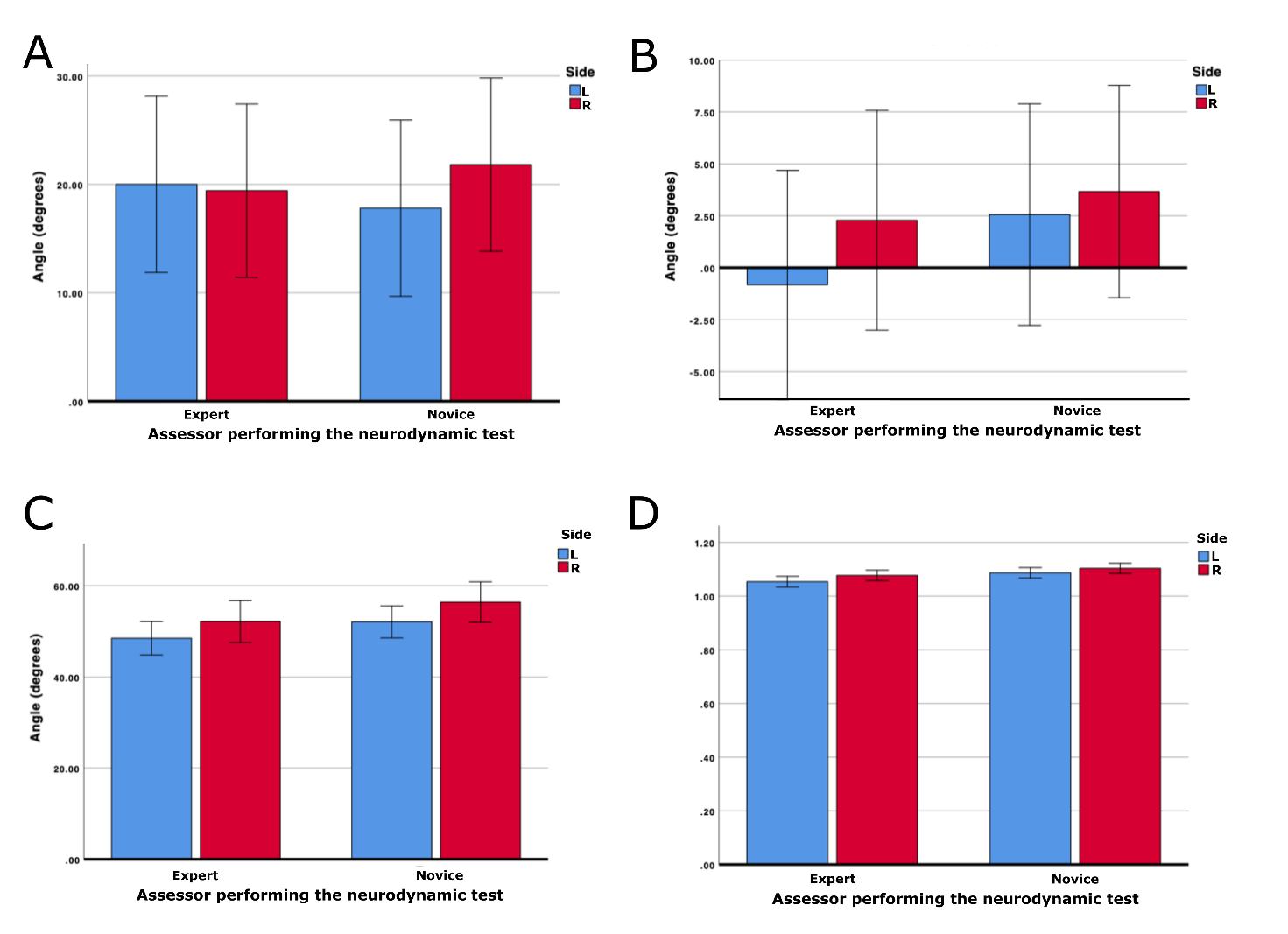

Supplement: Supplementary file 1 [file Data_Sheet_1.docx]
